# Long-term trends of alanine aminotransferase levels among persons living with human immunodeficiency virus/hepatitis B virus with and without hepatitis delta coinfection

**DOI:** 10.3389/fmed.2022.988356

**Published:** 2022-09-15

**Authors:** Lorin Begré, Charles Béguelin, Anders Boyd, Lars Peters, Jürgen Rockstroh, Huldrych F. Günthard, Enos Bernasconi, Matthias Cavassini, Karine Lacombe, Amanda Mocroft, Gilles Wandeler, Andri Rauch

**Affiliations:** ^1^Department of Infectious Diseases, Inselspital, Bern University Hospital, University of Bern, Bern, Switzerland; ^2^Graduate School for Health Sciences, University of Bern, Bern, Switzerland; ^3^Department of Infectious Diseases, Research and Prevention, Public Health Service of Amsterdam, Stichting HIV Monitoring, Amsterdam, Netherlands; ^4^CHIP, Rigshospitalet, University of Copenhagen, Copenhagen, Denmark; ^5^HIV Clinic, Department of Medicine, University Hospital Bonn, Bonn, Germany; ^6^Department of Infectious Diseases, University Hospital Zurich, Zurich, Switzerland; ^7^Institute of Medical Virology, University of Zurich, Zurich, Switzerland; ^8^Division of Infectious Diseases, Regional Hospital Lugano EOC, University of Geneva and University of Southern Switzerland, Lugano, Switzerland; ^9^Division of Infectious Diseases, University Hospital Lausanne, University of Lausanne, Lausanne, Switzerland; ^10^INSERM IPLESP, St Antoine Hospital, AP-HP, Sorbonne Université, Paris, France; ^11^Center for Clinical Research, Epidemiology, Modeling, and Evaluation, Institute for Global Health, University College London, London, United Kingdom

**Keywords:** hepatitis D (delta) virus, hepatitis B virus, HIV, coinfection, tenofovir, alanine aminotransferase elevation

## Abstract

**Background:**

Hepatitis delta virus (HDV) infection accelerates the progression of liver disease in persons living with HIV and hepatitis B virus (HBV) coinfection. We explored the association between HDV infection and alanine aminotransferase (ALT) elevation during tenofovir-containing antiretroviral treatment among persons living with HIV/HBV.

**Materials and methods:**

We included persons living with HIV/HBV with and without HDV starting tenofovir-containing antiretroviral therapy (ART) in three European cohorts with at least 18 months of follow-up. We defined HDV infection as a positive anti-HDV antibody test. We assessed risk factors for ALT elevation ≥ 1.25x upper limit of normal after 5 years of tenofovir-treatment using multivariate logistic regression models. The difference in ALT trends between individuals with and without HDV was evaluated using linear mixed effects models.

**Results:**

61/518 (11.8%) participants had an HDV infection. Among individuals with HDV, 63.9% had ALT elevation after 2 years and 55.6% after 5 years of tenofovir, whereas the estimates were 34.1% after two and 27.0% after 5 years in those without HDV. HDV coinfection (adjusted odds ratio 2.8, 95% confidence interval 1.4–5.8) and obesity at baseline (adjusted odds ratio 3.2, 95% confidence interval 1.2–8.0) were associated with ALT elevation after 5 years of tenofovir therapy. Mean ALT levels were consistently higher during follow-up in participants with HDV compared to those without HDV.

**Conclusion:**

Persistent ALT elevation is common in persons living with HIV/HBV in Europe despite adequate HBV therapy. HDV coinfection and obesity are independent risk factors for persistent ALT elevation during long-term tenofovir treatment.

## Introduction

Hepatitis B virus (HBV) infection is a major cause of morbidity and mortality among persons living with HIV (PLWH) (1). Of the approximately 38 million PLWH, an estimated 8% are also living with hepatitis B (2, 3). Hepatitis delta (HDV) coinfection occurs in approximately 15% of persons living with HIV/HBV in Europe and the majority of them have detectable HDV ribonucleic acid (RNA) (4). Currently, a large majority of persons living with HIV/HBV/HDV or HIV/HBV are treated with tenofovir disoproxil fumarate (TDF) or tenofovir alafenamide (TAF) as part of their antiretroviral treatment (ART). This treatment suppresses HBV viral load successfully but the risk of liver inflammation, liver-related events and death remains elevated (5). In a recent analysis from the Swiss HIV Cohort Study, the risk of liver-related death was eight times higher among individuals with HDV infection compared to those without (6).

Tenofovir leads to liver fibrosis regression, and early alanine aminotransferase (ALT) normalization after initiation of HBV treatment is associated with lower risk for hepatocellular carcinoma in persons living with hepatitis B (7, 8). In PLWH, the effect of tenofovir on liver fibrosis regression appears to be smaller and ALT elevation was identified as an independent risk factor for advanced fibrosis (9). Data on long-term trends of ALT levels among large populations of persons living with HIV/HBV are scarce. Understanding the risk factors leading to persistent ALT elevation despite adequate HBV therapy including the impact of HDV could help reduce the risk for liver-related events in this population.

The objective of this study was to explore the association between HDV infection and long-term trends in ALT levels after initiation of tenofovir-containing ART in the Euro-B study, a multi-cohort collaboration including persons living with HIV/HBV and with HIV/HBV/HDV from the Swiss HIV Cohort Study (10), the EuroSIDA Study (11), and the French HIV/HBV cohort (12).

## Materials and methods

### Study design and population

We included all PLWH aged 18 years or older with two positive HBsAg measurements ≥ 6 months apart who started a tenofovir disoproxil fumarate or tenofovir alafenamide-containing ART between November 2001 and September 2019 and had at least two available ALT measurements, one at the start and the other 24 months after start of TDF or TAF treatment. We excluded participants without known HDV serology. Participants could switch from TDF to TAF or vice versa during follow-up. Detailed information on demographical, clinical, and laboratory data were collected according to the standardized study protocols of the Swiss HIV Cohort Study, the EuroSIDA Study and the French HIV/HBV cohort (10–12). Local ethical committees approved the cohort studies and written consent was obtained from all participants according to local regulations.

### Outcomes and definitions

Our primary outcome was the proportion of participants with an ALT elevation ≥ 1.25x upper limit of normal (ULN) 2 and 5 years after start of tenofovir treatment in persons with and without HDV coinfection. Our secondary outcome was the difference in mean ALT levels from tenofovir start to 5 years thereafter. We defined ALT ULN as 35 international units per liter (IU/L) for men and 25 IU/L for women according to the AASLD definition (13). We defined mild ALT elevation as ALT ≥ 1.25x to < 2.5x ULN, moderate ALT elevation as ≥ 2.5x to < 5x ULN, severe ALT elevation as ALT ≥ 5x to < 10x ULN and life-threatening ALT elevation as ≥ 10x ULN as proposed by the National Institutes of Health’s Division of AIDS (14).

We classified participants with a positive anti-hepatitis delta antibody (anti-HDV) test at any time point as having HDV coinfection. We defined HBV viral load detection limit as 20 international units per milliliter (IU/ml) or the detection limit reported. Participants were considered to be hepatitis C virus (HCV) RNA positive if HCV RNA was quantifiable before tenofovir start. We defined liver cirrhosis primarily according to results from liver biopsy. If no liver biopsy was performed, we used a liver stiffness measurement > 11 kilopascal (kPa) using transient elastography or aspartate aminotransferase (AST)-to-platelet ratio (APRI) index > 2 to classify participants (15, 16). We considered reporting of ascites, bleeding from gastric esophageal varices, portal hypertension, hepatic encephalopathy, spontaneous bacterial peritonitis, and histologically confirmed diagnosis of cirrhosis, hepatorenal syndrome and liver transplantation as liver-related events. As alcohol consumption was not uniformly assessed across all cohorts, we harmonized the data and used an intake of > 25 alcohol containing units per week for men and > 20 alcohol containing units per week for women to define unhealthy alcohol use. We defined diabetes mellitus as reported diagnosis of diabetes mellitus or treatment with a blood glucose lowering drug; hypertension as reported diagnosis of arterial hypertension or treatment with an antihypertensive drug; and dyslipidemia as a total cholesterol to HDL-cholesterol ratio > 5 or treatment with a lipid lowering drug.

### Statistical analysis

We defined baseline as the start date of the first tenofovir-containing ART. For the assessment of the proportion of participants with ALT elevation after two and 5 years of tenofovir-containing ART, we considered the closest measurements to baseline (−12/ + 6 months), to 24 months (± 6 months), and to 60 months (± 6 months) of tenofovir treatment. For the longitudinal assessment of ALT levels, all available ALT measurements from the closest laboratory measurement to baseline (−12/ + 6 months) up to 60 months (+ 6 months) afterward were considered. Follow-up was censored at death, loss to follow-up, last follow-up visit or 6 months after cessation of the last tenofovir-containing drug, whichever happened first. Participants interrupting tenofovir treatment were allowed to continue follow-up if they resumed treatment later on.

We compared demographic and clinical characteristics at baseline between participants with and without HDV using Pearson’s chi-squared tests for categorical variables and Wilcoxon rank-sum tests for continuous variables. We assessed the proportion of participants with at least mild ALT elevation after 2 and 5 years. We used multivariable logistic regression to analyze potential risk factors for ALT elevation after two and after 5 years of tenofovir treatment. For the multivariable model, we included all variables with a *p*-value < 0.1 in univariable analyses, but excluded mode of HIV acquisition due to collinearity with HDV status.

We modeled mean ALT values with 95% confidence intervals (CI) over time using multivariable linear mixed effect models with a random intercept for individuals and a random slope for individual follow-up time. We included HDV status as a covariate to compare mean ALT levels between participants with and without HDV. We incorporated follow-up time as restricted cubic splines with four knots located at the 5th, 35th, 65th, and 95th percentile. We based 95% CI calculation on standard errors calculated using the delta method. We adjusted our multivariable model for sex to control for biological differences in ALT levels between males and females, and for ART experience at tenofovir start to control for potential immune reconstitution-induced hepatic flares. Treatment with TDF and TAF were included as separate time-updated covariates with an interaction term between them to take into account treatment interruptions and the potential additional beneficial impact of TAF on ALT values (17). In addition, we included all baseline variables with a *p*-value < 0.1 in univariable analyses of risk factors for ALT elevation after 2 and 5 years of tenofovir treatment in a preliminary model but excluded those with a *p*-value > 0.1 in a backward stepwise fashion from the final model. In the final model, BMI was included as a time-updated covariate rather than BMI at baseline to control for the influence of weight changes on ALT levels over time. Missing BMI assessments at a specific data point were handled by carrying the last observation forward. Missing values of categorical baseline covariates were included as a separate category. In a sub-analysis, we investigated the impact of HBV-active nucleoside reverse transcriptase inhibitor (NRTI) pretreatment on ALT levels in participants with and without HDV.

In sensitivity analyses, we ran the multivariable models using detectable HDV RNA at any time point instead of a positive anti-HDV test as the definition of HDV infection. Statistical significance was defined as a two-sided *p*-value < 0.05. We performed all analyses using Stata/MP 16.1 (StataCorp, College Station, TX, United States).

## Results

### Study population

We identified 614 participants with chronic hepatitis B starting TDF or TAF, of whom we excluded 35 without available ALT measurements at tenofovir start and after 24 months. We further excluded 61 participants with unknown HDV serology. In total, we included 518 participants with a median follow-up time of 9.1 years [interquartile range (IQR) 5.6–13.3] after initiation of the first tenofovir-containing regimen. Excluded participants did not differ significantly from the included study population with regards to age, BMI, mode of HIV acquisition, liver cirrhosis, and hepatitis B e antigen (HBeAg) status, but they were less likely to be treated with an HBV-active NRTI prior to the initiation of tenofovir [55/96 (57.3%) vs. 387/518 (74.7%), *p* < 0.001].

Hepatitis delta virus (HDV) serology was positive in 61 (11.8%) participants. The characteristics of participants with and without HDV coinfection at start of tenofovir therapy are shown in Table 1. Participants with HDV coinfection were more likely to have acquired HIV through injection drug use (62.3% vs. 5.9%, *p* < 0.001), to have HCV replication (25.9% vs. 4.1%, *p* < 0.001), to be of European origin (82.0% vs. 65.1%, *p* = 0.01) and to have liver cirrhosis (29.4% vs. 11.0%, *p* = 0.002), but were less likely to have a detectable HBV viral load (55.6% vs. 75.9%, *p* = 0.003), and to be HBeAg-positive (31.0% vs. 54.3%, *p* = 0.004). Of 42 participants with HDV coinfection and available HDV viral load quantification, 26 (61.9%) had detectable HDV RNA. Median HDV viral load was 11,930,000 copies/ml (IQR 170,284 to 129,862,224) among participants with detectable HDV RNA and HDV genotyping was available in 18 of them with HDV genotype 1 being predominant (94.4%).

**TABLE 1:** Characteristics of Euro-B participants at start of tenofovir-containing antiretroviral therapy (ART), by anti-hepatitis delta antibody (anti-HDV) status.

	anti-HDV negative	anti-HDV positive	*p*-value
	*N* = 457	*N* = 61	
Median age in years (IQR)	41 (36–47)	40 (34–44)	0.08
Median calendar year of tenofovir start (IQR)	2005 (2003–2008)	2005 (2003–2007)	0.73
Median follow-up time in years (IQR)	9.1 (5.6–13.1)	10.0 (5.6–15.0)	0.47
Female sex	81/457 (17.7%)	17/61 (27.9%)	0.06
Mode of HIV acquisition			< 0.001
men who have sex with men	256/457 (56.0%)	8/61 (13.1%)	
heterosexual	119/457 (26.0%)	13/61 (21.3%)	
injection drug use	27/457 (5.9%)	38/61 (62.3%)	
other or unknown	55/457 (12.0%)	2/61 (3.3%)	
European origin	293/450 (65.1%)	50/61 (82.0%)	0.01
CDC stage C	122/457 (26.7%)	18/61 (29.5%)	0.64
Liver cirrhosis	38/344 (11.0%)	10/34 (29.4%)	0.002
Ever reported unhealthy alcohol use	101/436 (23.2%)	19/57 (33.3%)	0.09
Diabetes mellitus	15/457 (3.3%)	0/61 (0.0%)	0.15
Hypertension	59/457 (12.9%)	8/61 (13.1%)	0.96
Dyslipidemia	181/432 (41.9%)	24/60 (40.0%)	0.78
BMI ≥ 30 kg/m^2^	27/432 (6.3%)	2/59 (3.4%)	0.38
ART-experienced	293/457 (64.1%)	36/61 (59.0%)	0.44
Pretreatment with HBV-active NRTI*	341/457 (74.6%)	46/61 (75.4%)	0.89
ALT ≥ 1.25x ULN	227/457 (49.7%)	39/61 (63.9%)	0.04
Detectable HBV viral load	289/381 (75.9%)	25/45 (55.6%)	0.003
HBeAg positive	185/341 (54.3%)	13/42 (31.0%)	0.004
CD4 ≥ 500 cells/μl	128/455 (28.1%)	10/61 (16.4%)	0.05
Detectable HIV viral load	231/453 (51.0%)	33/61 (54.1%)	0.65
Hepatitis C RNA positive	17/416 (4.1%)	14/54 (25.9%)	< 0.001
HDV RNA positive	−	26/42 (61.9%)	−

Data are presented as median (IQR) for continuous measures, and n/total (%) for categorical measures. *424 participants received lamivudine and 1 received entecavir prior to tenofovir. 17 participants who received lamivudine received also adefovir and 2 entecavir prior to tenofovir. ALT, alanine aminotransferase; anti-HDV, anti-hepatitis delta antibodies; ART, antiretroviral therapy; BMI, body mass index; CDC, centers for disease control and prevention; HBeAg, hepatitis B e antigen; HBV, hepatitis B virus; HIV, human immunodeficiency virus; IQR, interquartile range; NRTI, nucleoside reverse transcriptase inhibitors; ULN, upper limit of normal; RNA, ribonucleic acid.

425/518 (82.0%) participants were followed for at least 5 years on tenofovir treatment and 401 (94.4%) of them had an available ALT measurement after 60 months. Of the 93 participants with less than 5 years of follow-up on tenofovir, 71 participants reached their last follow-up visit earlier or were lost to follow-up, 14 participants died and eight stopped tenofovir treatment permanently. Within the 5°year follow-up period, 15/401 (3.7%) participants had switched from TDF to TAF and 87/401 (21.7%) had interrupted tenofovir treatment for > 90 days cumulatively. Proportions of treatment interruptions did not differ between HDV negative and HDV positive participants. 12/401 (3.0%) participants received treatment for HCV.

### Alanine aminotransferase elevation during five years of tenofovir treatment

**FIGURE 1:**
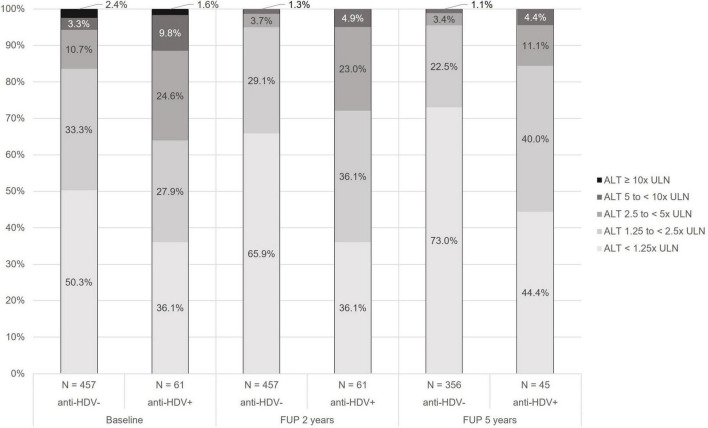
Grade of alanine aminotransferase (ALT) elevation at start, after 2 years and after 5 years of tenofovir treatment by anti-hepatitis delta antibody (anti-HDV) status. Grading according to the National Institutes of Health’s Division of acquired immunodeficiency syndrome (AIDS) (14). ALT, alanine aminotransferase; anti-HDV, anti-hepatitis delta antibodies; FUP, follow-up; ULN, upper limit of normal.

At start of tenofovir therapy, 227/457 (49.7%) HDV-negative participants had at least mildly elevated ALT, compared to 39/61 (63.9%) HDV-positive participants (*p* = 0.04). 26 (5.7%) participants without HDV had severe or life-threatening ALT elevation at start of tenofovir treatment compared to 7 (11.5%) with HDV coinfection (Figure 1). A sensitivity analysis classifying HDV coinfection as having detectable HDV RNA showed similar results with an even higher proportion of participants with at least mild ALT elevation in the group with detectable HDV RNA (84.6 vs. 49.3%, *p* < 0.001) (Supplementary Table 1). After 5 years of treatment, the proportion of participants with at least mildly elevated ALT decreased to 96/356 (27.0%) of HDV negative participants and 25/45 (55.6%) of the participants with HDV coinfection. Severe ALT elevation was observed in 4/356 (1.1%) HDV negative and 2/45 (4.4%) HDV positive participants (Figure 1).

In multivariable analyses, HDV coinfection was associated with ALT elevation after two [adjusted odds ratio (aOR) 5.6, 95% CI 2.6–12.4] and 5 years (aOR 2.8, 95% CI 1.4–5.8) (Table 2 and Supplementary Table 2). ALT at baseline (aOR 2.3, 95% CI 1.4–3.8), younger age (aOR 1.0, 95% CI 0.9–1.0), and obesity at baseline (aOR 3.2, 95% CI 1.2–8.0) were significantly associated with elevated ALT after 5 years of tenofovir treatment (Table 2). In a sensitivity analysis using HDV RNA instead of anti-HDV to define HDV coinfection, the multivariable models showed similar results compared to the main model: HDV RNA was strongly associated with at least mild ALT elevation after two (aOR 13.2, 95% CI 2.9–59.7) and 5 years (aOR 4.2, 95% CI 1.4–12.5) (Supplementary Table 3).

**TABLE 2:** Risk factors at start of tenofovir-containing antiretroviral therapy (ART) for alanine aminotransferase (ALT) elevation (≥ 1.25x ULN) after 5 years of tenofovir treatment.

	Unadjusted		Adjusted	
	OR (95% CI)	*P*-value	OR (95% CI)^†^	*P*-value
Anti-HDV status				
negative	1.0	(ref)	1.0	(ref)
positive	3.4 (1.8–6.4)	< 0.001	2.8 (1.4–5.8)	0.005
ALT at baseline				
< 1.25x ULN	1.0	(ref)	1.0	(ref)
≥ 1.25x ULN	2.4 (1.5–3.7)	< 0.001	2.3 (1.4–3.8)	0.001
Age [years]	1.0 (0.9–1.0)	0.01	1.0 (0.9–1.0)	0.02
Female sex	1.2 (0.7–2.0)	0.54		
Mode of HIV acquisition		0.005		
men who have sex with men	1.0			
heterosexual	1.0 (0.6–1.8)			
injection drug use	3.1 (1.6–5.9)			
other or unknown	1.0 (0.5–2.2)			
Liver cirrhosis	1.7 (0.8–3.5)	0.14		
History of liver related event	2.4 (1.0–5.8)	0.04	2.1 (0.7–5.9)	0.18
Ever reported unhealthy alcohol use	1.2 (0.8–2.0)	0.41		
Dyslipidemia	1.2 (0.8–1.8)	0.50		
Diabetes mellitus	1.3 (0.4–4.6)	0.65		
Hypertension	1.2 (0.6–2.2)	0.59		
BMI ≥ 30 kg/m^2^	2.4 (1.0–5.7)	0.05	3.2 (1.2–8.0)	0.02
HBV-active NRTI pretreatment	1.1 (0.7–1.8)	0.70		
ART-experienced	1.4 (0.9–2.1)	0.19		
Detectable HBV viral load	1.0 (0.6–1.7)	0.96		
HCV RNA positive	2.4 (1.0–5.6)	0.05	1.4 (0.5–3.8)	0.47

^†^347 participants included in complete case analysis. ALT, alanine aminotransferase; anti-HDV, anti-hepatitis delta antibodies; ART, antiretroviral therapy; BMI, body mass index; CI, confidence interval; FUP, follow-up; HBV, hepatitis B virus; HCV, hepatitis C virus; NRTI, nucleoside reverse transcriptase inhibitors; OR, odds ratio; RNA, ribonucleic acid.

Hepatitis delta virus-positive individuals were less likely to have a detectable HBV viral load compared to HDV-negative individuals at baseline [25/45 (55.6%) vs. 289/381 (75.9%), *p* = 0.003], and after 5 years of follow-up [0/18 (0.0%) vs. 25/204 (12.3%), *p* = 0.11]. Of note, HDV-negative participants with replicating HBV infection 5 years after tenofovir start were more likely to have elevated ALT compared to those with suppressed HBV viral load [12/50 (24%) vs. 13/154 (8.4%), *p* = 0.004].

### Longitudinal analysis of alanine aminotransferase levels over five years of tenofovir treatment

**FIGURE 2:**
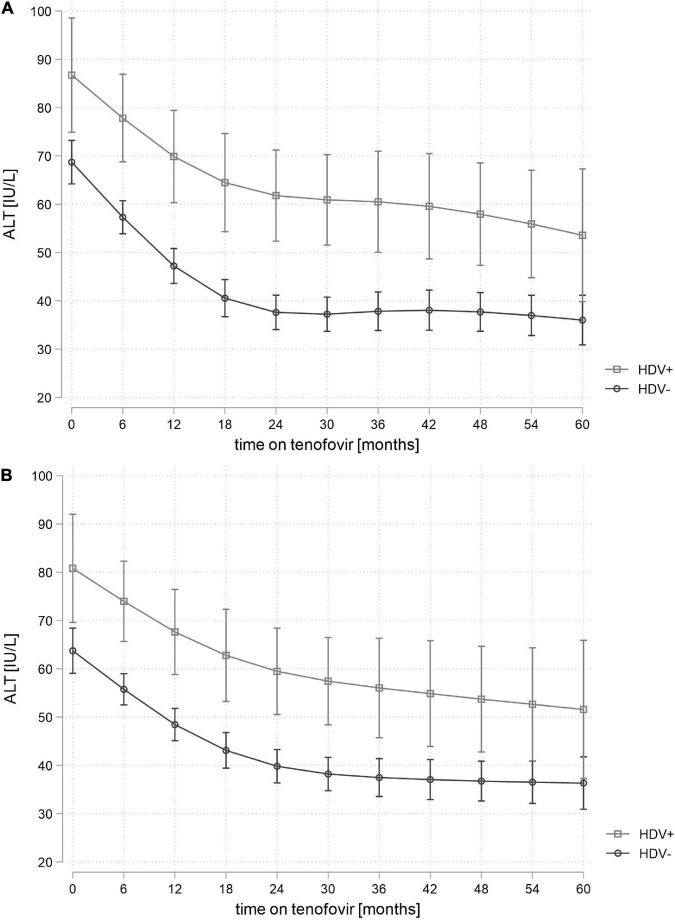
Unadjusted **(A)** and adjusted* **(B)** predicted mean alanine aminotransferase (ALT) values in participants with and without hepatitis delta virus (HDV) coinfection during treatment with tenofovir. Adjusted for ALT level, age, sex, detectable hepatitis B virus (HBV) viral load, hepatitis C virus (HCV) ribonucleic acid (RNA) status and antiretroviral therapy (ART)-experience at baseline, time-updated body mass index (BMI) and treatment with tenofovir prodrugs (tenofovir disoproxil fumarate or tenofovir alafenamide). ALT, alanine aminotransferase; ART, antiretroviral therapy; BMI, body mass index; HBV, hepatitis B virus; HCV, hepatitis C virus; HDV+, anti-hepatitis delta antibodies positive; HDV-, anti-hepatitis delta antibodies negative; IU/L, international units per liter; RNA, ribonucleic acid.

Alanine aminotransferase trends were assessed among 510 participants and included a total of 6,687 ALT measurements (median 11 measurements per participant, IQR 8–15). The difference in predicted mean ALT values between participants with and without HDV coinfection was + 17 IU/L (95% CI 5–29) at baseline, + 20 IU/L (95% CI 10–29) after 2 years and + 15 IU/L (95% CI 0–31) after 5 years of tenofovir treatment (Figure 2). In a sensitivity analysis using HDV RNA instead of anti-HDV to define HDV coinfection, the difference in mean ALT values between participants with and without HDV coinfection increased to + 29 IU/L (95% CI 12–46) at baseline, + 31 IU/L (95% CI 18–44) after 2 years and + 40 (95% CI 18–62) after 5 years of tenofovir treatment in the multivariable model (Supplementary Figure 1). HBV-active NRTI treatment before the initiation of tenofovir treatment was not associated with ALT trends in participants with and without HDV coinfection (Supplementary Figure 2).

## Discussion

In our multi-cohort study of persons living with HIV/HBV in Europe, over 30% of study participants had elevated ALT levels after 5 years of tenofovir therapy, this risk being three times higher in persons living with HDV coinfection. Younger age, obesity and ALT levels at the start of tenofovir therapy were also associated with ALT elevation after 5 years. Our study highlights the need to identify and address risk factors for persistent liver inflammation among persons living with HIV/HBV, particularly in those with HDV coinfection.

During 5 years of tenofovir therapy, ALT levels were persistently higher among persons with HDV coinfection compared to those without HDV. In the subgroup of participants with quantifiable HDV RNA, the difference in ALT levels compared to HDV negative individuals was even more accentuated. Our findings correspond with the results of a recent study in HIV-negative individuals with HBV treatment in Taiwan, in which HDV RNA positivity was found to be the strongest factor associated with ALT elevation after 2 years of NRTI therapy (18). ALT elevation in HDV positive individuals during treatment could be explained by the marginal impact of tenofovir on HDV replication, despite its efficacy in suppressing HBV viral load (19, 20). Persistent HDV replication contributes to chronic liver inflammation and leads to high rates of liver decompensation and death in persons living with HIV/HBV/HDV (21). A recent study in persons living with HIV/HBV from Italy observed a doubling of the risk for a composite outcome of liver-related events, liver-related death, and non-invasive assessment of cirrhosis in individuals with HDV coinfection (22). Currently, HDV treatment options remain limited for these patients, despite the recent approval of bulevirtide (23). Our study demonstrates that persistent liver inflammation occurs in a substantial number of PLWH with HDV despite optimal HBV therapy, which underscores the need for HDV testing and liver disease monitoring in all persons living with chronic hepatitis B (24, 25).

We found higher rates of ALT elevation in HDV-negative persons living with HIV/HBV compared to published data from HIV-negative persons with HBV: in an analysis of 471 individuals from Europe, North America, Australia, and New-Zealand who participated in two randomized controlled trials initially assessing the antiviral efficacy of TDF in comparison to adefovir, less than 20% had ALT elevation after 5 years of TDF therapy (26). However, comparison across studies is limited by the differences in clinical and sociodemographic characteristics, as well as in treatment eligibility criteria in the presence of HIV infection (24, 25). A recent study among adults living with HIV and HBV found histologic evidence of fatty liver disease in 30% of persons, which was associated with elevated ALT over time (27). In our study, the presence of obesity increased the risk for ALT elevation after 5 years of tenofovir therapy. ALT elevation seems to be common among persons living with HIV and HBV in absence of HDV coinfection, which highlights the need to address and appropriately treat metabolic risk factors for liver inflammation and fibrosis among all PLWH (24).

Our study provides detailed information on ALT levels over time from a large cohort of persons living with HIV and HBV across Europe. Our strict inclusion criteria and comprehensive clinical and virological data allowed us to obtain robust estimates for the association between HDV infection and liver inflammation. With detailed treatment histories available, we were able to disentangle the impact of HBV treatment prior to tenofovir, ART as well as metabolic and infectious comorbidities on ALT levels. However, given the limited number of participants treated with TAF in our study, we were not able to assess if long-term ALT trends depended on the type of tenofovir prodrug used (28). Hepatitis serologies and data on medical history like alcohol consumption were assessed according to the specific protocols of the participating cohorts, and were not always collected uniformly. Furthermore, some data on covariates were missing, as depicted in Table 1. However, the bias introduced should be small as the amount of missing values was similar in HDV positive and negative participants except for the assessment of liver cirrhosis. As HDV status was not assessed systematically at start of tenofovir therapy and serial HDV assessments were not available, HDV-positive individuals may have been at different stages of HDV infection at start of tenofovir therapy. In addition, we cannot differentiate participants living with HIV/HBV/HDV at start of tenofovir therapy from those acquiring HDV as superinfection during the study period. This could have led to an underestimation of the difference in ALT levels in case of a participant classified as HDV negative newly acquiring HDV after starting tenofovir therapy.

In summary, coinfection with hepatitis delta was an independent risk factor for persistent ALT elevation during long-term tenofovir treatment in persons living with HIV/HBV. Furthermore, obesity was independently associated with higher ALT levels over time. Careful monitoring of ALT elevations and liver disease progression is recommended in persons living with HIV/HBV, particularly in those with HDV coinfection or other comorbidities leading to liver inflammation.

## Data availability statement

The data analyzed in this study is subject to the following licenses/restrictions: For open data sharing, the data is too dense and comprehensive to preserve patient privacy in persons living with HIV. The participating cohorts can be approached for data requests. Requests to access these datasets should be directed to http://www.shcs.ch/contact for the Swiss HIV Cohort Study and eurosida.rigshospitalet@regionh.dk for EuroSIDA.

## Ethics statement

The studies involving human participants were reviewed and approved by Kantonale Ethikkommission Bern and other national ethical committees from the different cohort sites (for the Swiss HIV Cohort Study: https://shcs.ch/206-ethic-committee-approval-and-informed-consent, for EuroSIDA: https://chip.dk/Research/Studies/EuroSIDA/Study-documents). The patients/participants provided their written informed consent to participate in this study.

## Author contributions

LB, CB, GW, and AR conceived the study. LB analyzed the data. LB, AB, GW, and AR wrote the first draft of the manuscript. All authors collected and provided data for the study, reviewed and commented on the draft, and approved the final version.
