# Supplementary material for: Long-term trends of alanine aminotransferase levels among persons living with human immunodeficiency virus/hepatitis B virus with and without hepatitis delta coinfection
Source: Front Med (Lausanne). 2022 Sep 15;9:988356. doi: 10.3389/fmed.2022.988356 (PMC9522477; doi:10.3389/fmed.2022.988356)
Supplement: Supplementary file 1 [file Data_Sheet_1.pdf]

Supplementary Material

Supplementary Table 1. Characteristics of Euro-B participants at start of tenofovir-containing antiretroviral therapy (ART), by hepatitis delta virus (HDV) replication status

	**HDV RNA negative N = 473**	**HDV RNA positive N = 26**	**P-value**
Median age in years (IQR)	41 (36-47)	36 (31-41)	0.004
Median calendar year of tenofovir start (IQR)	2005 (2003-2008)	2006 (2004-2009)	0.16
Median follow-up time in years (IQR)	9.1 (5.7-13.2)	10.1 (5.6-13.7)	0.84
Female sex	87/473 (18.4%)	6/26 (23.1%)	0.55
Mode of HIV acquisition			< 0.001
men who have sex with men	259/473 (54.8%)	1/26 (3.8%)	
heterosexual	125/473 (26.4%)	5/26 (19.2%)	
injection drug use	34/473 (7.2%)	19/26 (73.1%)	
other or unknown	55/473 (11.6%)	1/26 (3.8%)	
European origin	304/466 (65.2%)	22/26 (84.6%)	0.04
CDC stage C	129/473 (27.3%)	5/26 (19.2%)	0.37
Liver cirrhosis	39/351 (11.1%)	6/18 (33.3%)	0.005
Ever reported unhealthy alcohol use	104/451 (23.1%)	8/25 (32.0%)	0.31
Diabetes mellitus	15/473 (3.2%)	0/26 (0.0%)	0.36
Hypertension	60/473 (12.7%)	4/26 (15.4%)	0.69
Dyslipidemia	187/448 (41.7%)	11/26 (42.3%)	0.95
BMI ≥ 30 kg/m^2	28/447 (6.3%)	1/26 (3.8%)	0.62
ART-experienced	303/473 (64.1%)	14/26 (53.8%)	0.29
Pretreatment with HBV-active NRTI	354/473 (74.8%)	17/26 (65.4%)	0.28
ALT ≥ 1.25x ULN	233/473 (49.3%)	22/26 (84.6%)	< 0.001
Detectable HBV viral load	296/395 (74.9%)	13/19 (68.4%)	0.52
HBeAg positive	189/354 (53.4%)	6/19 (31.6%)	0.06
CD4 ≥ 500 cells/µl	129/471 (27.4%)	6/26 (23.1%)	0.63
Detectable HIV viral load	238/469 (50.7%)	16/26 (61.5%)	0.28
Hepatitis C RNA positive	21/431 (4.9%)	4/22 (18.2%)	0.01

Data are presented as median (IQR) for continuous measures, and n/total (%) for categorical measures. *Abbreviations: ALT: alanine aminotransferase, HDV: hepatitis delta, ART: antiretroviral therapy, BMI: body mass index, CDC: centers for disease control and prevention, HBeAg: hepatitis B e antigen, HBV: hepatitis B virus, HIV: human immunodeficiency virus, IQR: interquartile range, NRTI: nucleoside reverse transcriptase inhibitors, ULN: upper limit of normal, RNA: ribonucleic acid.*

Supplementary Table 2. Risk factors at start of tenofovir-containing antiretroviral therapy (ART) for alanine aminotransferase (ALT) elevation (≥ 1.25x ULN) after two years of tenofovir treatment

	Unadjusted OR (95% CI)	P-value	Adjusted OR (95% CI)†	P-value
Anti-HDV status				
negative	1.0	(ref)	1.0	(ref)
positive	3.4 (2.0-6.0)	< 0.001	5.6 (2.6-12.4)	< 0.001
ALT at baseline				
< 1.25x ULN	1.0	(ref)	1.0	(ref)
≥ 1.25x ULN	3.2 (2.2-4.6)	< 0.001	2.1 (1.4-3.4)	0.001
Age [years]	1.0 (1.0-1.0)	0.36		
Female sex	0.8 (0.5-1.2)	0.26		
Mode of HIV acquisition		0.14		
men who have sex with men	1.0			
heterosexual	1.0 (0.6-1.5)			
injection drug use	1.8 (1.0-3.1)			
other or unknown	0.9 (0.5-1.6)			
Liver cirrhosis	0.9 (0.5-1.7)	0.77		
History of liver related event	1.6 (0.7-3.5)	0.28		
Ever reported unhealthy alcohol use	1.6 (1.1-2.5)	0.02	1.5 (0.9-2.5)	0.13
Dyslipidemia	1.1 (0.8-1.6)	0.54		
Diabetes mellitus	0.8 (0.3-2.4)	0.73		
Hypertension	1.1 (0.6-1.8)	0.83		
BMI ≥ 30 kg/m^2	1.0 (0.5-2.1)	0.95		
HBV-active NRTI pretreatment	1.5 (1.0-2.2)	0.08	1.1 (0.5-2.3)	0.80
ART-experienced	1.6 (1.1-2.3)	0.02	1.4 (0.7-2.7)	0.30
Detectable HBV viral load	1.6 (1.0-2.6)	0.04	1.9 (1.1-3.3)	0.02
Hepatitis C RNA positive	2.1 (1.0-4.4)	0.05	1.5 (0.5-4.5)	0.51

†379 participants included in complete case analysis. *Abbreviations: ALT: alanine aminotransferase, anti-HDV: anti-hepatitis delta antibodies, ART: antiretroviral therapy, BMI: body mass index, CI: confidence interval, HBV: hepatitis B virus, HCV: hepatitis C virus, NRTI: nucleoside reverse transcriptase inhibitors, OR: odds ratio, RNA: ribonucleic acid*

Supplementary Table 3. Sensitivity analysis of risk factors at start of tenofovir-containing antiretroviral therapy (ART) for alanine aminotransferase (ALT) elevation (≥ 1.25x ULN) after two and five years of tenofovir treatment with HDV RNA instead of anti-HDV to define hepatitis delta (HDV) coinfection. Only results from the multivariate analysis are shown (covariates from Table 2 and Supplementary Table 2 with P-value < 0.1 were included)

	**After 2 years**		**After 5 years**	
	**aOR (95% CI)**[†]	**P-value**	**aOR (95% CI)**[*]	**P-value**
HDV RNA status				
negative	1.0 (ref)		1.0 (ref)	
positive	13.2 (2.9-59.7)	0.001	4.2 (1.4-12.5)	0.01
ALT at baseline				
< 1.25x ULN	1.0 (ref)		1.0 (ref)	
≥ 1.25x ULN	1.9 (1.2-3.0)	0.01	2.2 (1.3-3.6)	0.002
Age [years]			1.0 (0.9-1.0)	0.02
History of liver related event			2.4 (0.8-7.1)	0.11
Ever reported unhealthy alcohol use	1.5 (0.9-2.4)	0.15		
BMI ≥ 30 kg/m^2			3.2 (1.3-8.2)	0.01
HBV-active NRTI pretreatment	1.2 (0.6-2.4)	0.66		
ART-experienced	1.3 (0.7-2.5)	0.44		
Detectable HBV viral load	1.8 (1.0-3.1)	0.05		
Hepatitis C RNA positive	1.8 (0.6-5.7)	0.30	1.7 (0.6-4.7)	0.27

[†]369 participants included in complete case analysis. [*]339 participants included in complete case analysis. *Abbreviations: ALT: alanine aminotransferase, aOR: adjusted odds ratio, ART: antiretroviral therapy, BMI: body mass index, CI: confidence interval, HBV: hepatitis B virus, HCV: hepatitis C virus, HDV: hepatitis delta virus, NRTI: nucleoside reverse transcriptase inhibitors, RNA: ribonucleic acid*

Supplementary Figure 1. Unadjusted **(A)** and adjusted* **(B)** predicted mean alanine aminotransferase (ALT) values in participants with and without hepatitis delta virus (HDV) replication during treatment with tenofovir

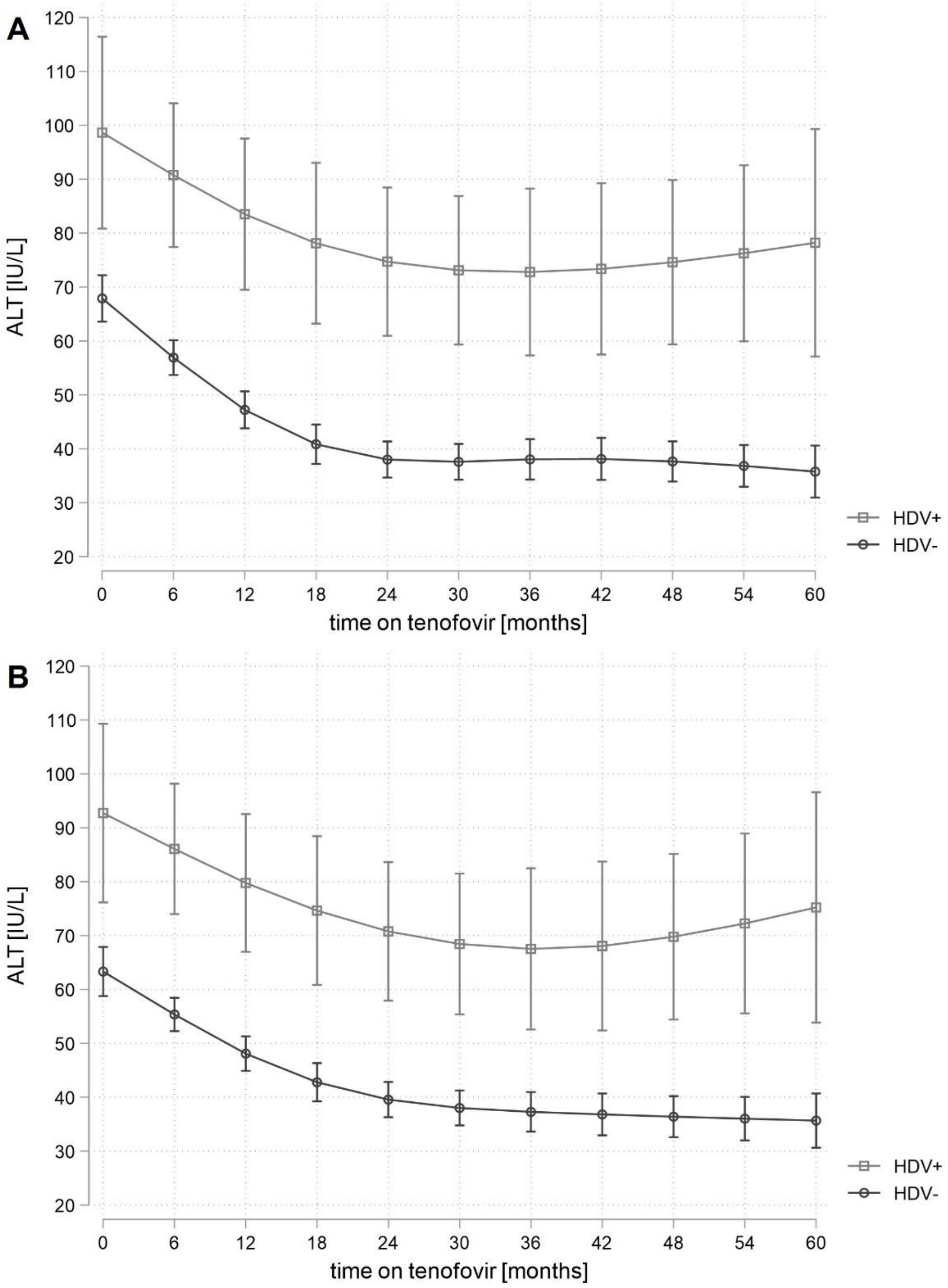

*Adjusted for ALT level, age, sex, detectable HBV viral load, HCV RNA status and ART-experience at baseline, time-updated BMI and time-updated treatment with tenofovir prodrugs (tenofovir disoproxil fumarate or tenofovir alafenamide). *ALT: alanine aminotransferase, ART: antiretroviral therapy, BMI: body mass index, HBV: hepatitis B virus, HCV: hepatitis C virus, HDV+: HDV RNA positive, HDV-: HDV RNA negative, IU/L: international units per liter, RNA: ribonucleic acid*

Supplementary Figure 2. Unadjusted **(A)** and adjusted* **(B)** predicted mean alanine aminotransferase (ALT) values in hepatitis B (HBV) treatment experienced† and HBV treatment naïve participants with and without hepatitits delta (HDV) during treatment with tenofovir

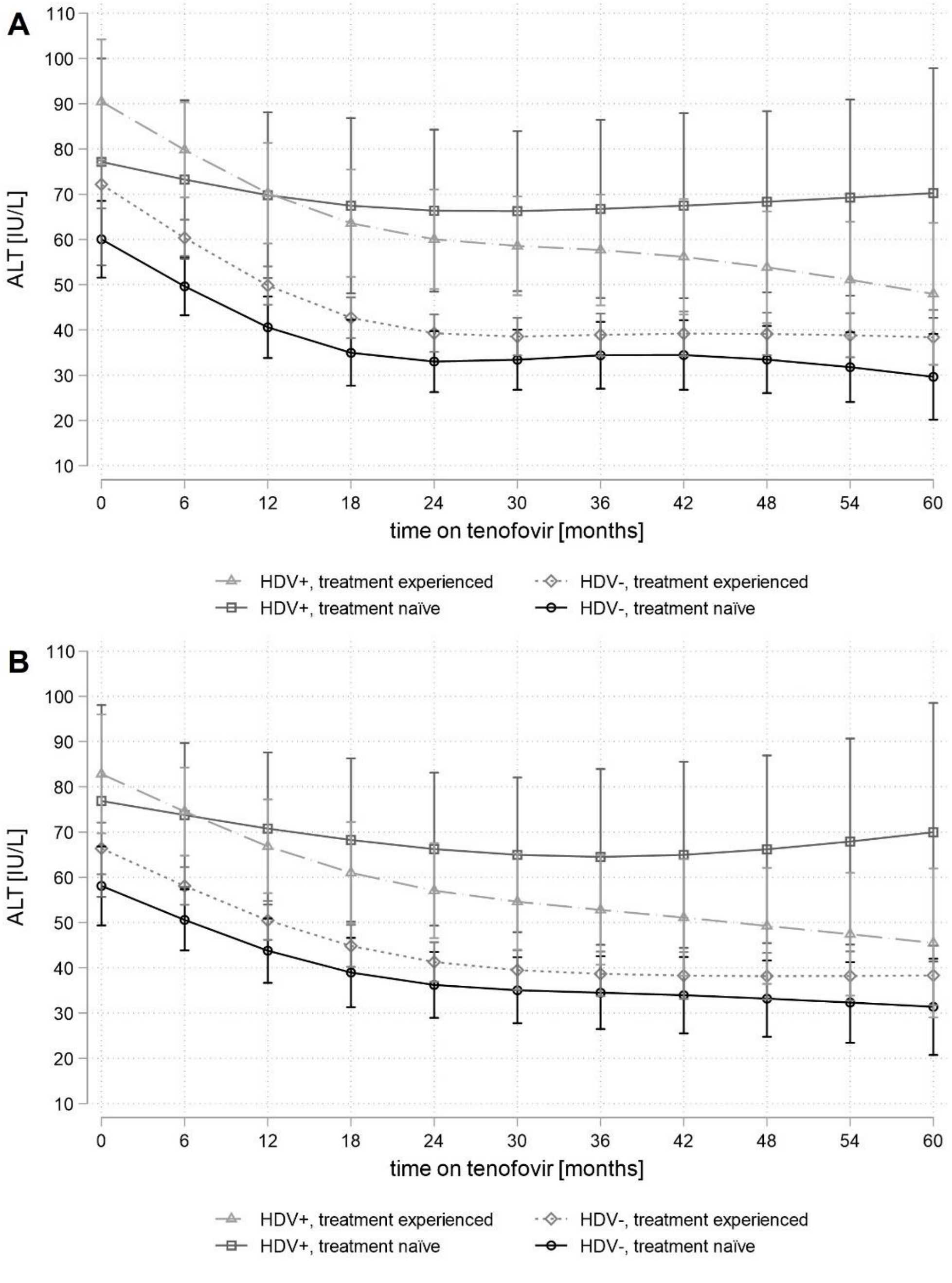

*Adjusted for ALT level, age, sex, detectable HBV viral load, HCV RNA status and ART-experience at baseline, time-updated BMI and treatment with tenofovir prodrugs (tenofovir disoproxil fumarate or tenofovir alafenamide), †treatment with lamivudine, adefovir or entecavir prior to tenofovir treatment. *ALT: alanine aminotransferase, ART: antiretroviral therapy, BMI: body mass index, HBV: hepatitis B virus, HCV: hepatitis C virus, HDV+: anti-hepatitis delta antibodies positive, HDV-: anti-hepatitis delta antibodies negative, IU/L: international units per liter, RNA: ribonucleic acid*
